# Reviews

**Published:** 1881-01

**Authors:** 


### Reviews.

**A Practical Treatise on the Diseases of Women.** By T. GAILLARD THOMAS, M. D., Professor of Diseases of Women, in the College of Physicians and Surgeons, New York. Fifth edition, enlarged and thoroughly revised, containing two hundred and sixty-six engravings on wood. Philadelphia: Henry C. Lea's Son & Co. 1880.

The accomplished author of this work, which has already secured a world-wide reputation, having been translated in several of the European languages, has devoted two years of labor in the endeavor to bring this edition (the fifth) to the level of the present state of the science of which it treats; and the enterprising publishers have also endeavored to place the work before the profession in such form as to render permanent its important contents. We are glad to take up a work of this character, bound in elegant half-Russia. Whatever Prof. Thomas writes in the special department of medicine, which owes so much to his ability and genius, does not need, however, an elegant and enriched envelop to commend it to the profession, but his medical writings deservedly claim the costly binding, that they may be placed in our library as one of the "classics" in modern medical literature.

We need not review the present edition only in so far as to refer to the valuable additions incorporated in this volume, and to commend it again to the profession. In the chapters devoted to uterine flexions, the lenient judgment of the reader is asked, until the measures recommended are tried. In this difficult class of displacements, the author shows the skill and ingenuity which has made him the most successful of American gynecologists. In the various devices he adopts, there is a practical and ingenious manner of reaching the desired end, that commends itself to our judgment, and in practice is uniformly successful.

The various subjects treated have been carefully reviewed, important additions made to bring them up to the present advanced state of our knowledge, and the entire work given the finishing touch, which the skillful hand and fertile resources of the author command. This volume has always been regarded the most valuable issued by the American profession. This edition is many times increased in value by the thorough revision and by the valuable additions it has received, and also by the elegant binding in which it is presented.

---

**Atlas of Human Anatomy.** Containing one hundred and eighty large plates, arranged according to Drs. Oesterreicher and Erdl, from their original designs from nature, and those of the greatest anatomists of modern times, viz: Weisse, Cloquet, Blumenbach, Langenbeck, Asch, Caldani, Meckel, &c., &c. With full and explanatory texts by J. A JEANCON. M. D. To be complete in forty-five parts, at seventy five cents each. A. E. Wilde & Co., Publishers, Cincinnati, O.

We have received of this valuable work parts I to X inclusive. Its object, as stated in the prospectus, is to bring before the public a pictorial illustration of all parts of the human body, in a size and form which ordinary works on anatomy fail to furnish. The size of the volume (22x35 in.) is ample to render every part illustrated in size and extent sufficiently clear to make the plate a most valuable guide to the student in the study of anatomy, and to the physician as an important work of reference.

As an example of the skill of the engraver, the plates are admirably executed, and reflect great credit upon the publishers for their enterprise in offering to the profession a work on anatomy superior in artistic finish, and in comprehensiveness of design, to any ever offered to the American profession.

We have examined with care each number as it has been received, and find the same precision in the execution of each plate as was noticeable in the initial number. We think the profession can well afford to second this undertaking, inasmuch as it supplies a want long felt. We shall take occasion to refer to this valuable work upon the reception of future numbers, and in the meantime hope that the publishers will be so far encouraged by the favor extended to their labor by the profession throughout the country, that they will succeed in their design to present the finest illustrations ever offered of the structure of the human body.

